# Severe and invasive bacterial infections in infants aged less than 90 days with and without SARS-CoV-2 infection

**DOI:** 10.1186/s13052-024-01721-x

**Published:** 2024-08-15

**Authors:** Giulia Brigadoi, Francesca Tirelli, Sara Rossin, Veronica Casotto, Francesca Riello, Giulia Gallinaro, Daniele Donà, Silvia Bressan, Liviana Da Dalt

**Affiliations:** 1 Department of Women’s and Children’ Health, Pediatric Infectious Disease Unit, Padua University, Padua, Italy; 2 Department of Women’s and Children’ Health, Pediatric Emergency Department, Padua University, Padua, Italy; 3 Department of Women’s and Children’ Health, Pediatric Rheumatology, Padua University, Padua, Italy

**Keywords:** Infants, Severe bacterial infection, SBI, Invasive bacterial infection, IBI, SARS-CoV-2 infection, COVID-19, Sepsis work-up

## Abstract

**Background:**

Fever in children represents one of the most common causes of medical evaluation. Infants younger than 90 days of age are at higher risk of severe and invasive bacterial infections (SBI and IBI). However, clinical signs and symptoms of viral and bacterial infections in young infants are frequently similar, and several studies have shown that the risk of SBIs remains non-negligible even in the presence of a positive point-of-care viral test. Our study aims to evaluate whether the proportion of SBIs and IBIs in febrile infants younger than 90 days during the COVID-19 pandemic was higher than that in the pre-pandemic period, and to describe the proportion of SBIs and IBIs in infants with and without SARS-CoV-2 infection.

**Methods:**

This was a retrospective single-center cohort study conducted at the Children's Hospital of the University of Padua in Italy, involving febrile young infants evaluated in the Pediatric Emergency Department (PED) and admitted to Pediatric Acute Care Unit (PACU) between March 2017 to December 2022. Infants admitted before the COVID-19 pandemic were compared to infants admitted during the pandemic period and SARS-CoV-2 positive patients to the negative ones.

**Results:**

442 febrile infants younger than 90 days were evaluated in Padua PED and admitted to the wards. The proportion of SBIs and IBIS did not significantly change over the study periods, ranging between 10.8% and 32.6% (*p* = 0.117) and between 0% and 7.6%, respectively (*p* = 0.367). The proportion of infants with a diagnosis of SBIs and IBIs was higher in the SARS-CoV-2 negative group (30.3% and 8.2%, respectively) compared to the positive group (8.5% and 2.8%, respectively) (*p* < 0.0001). The most common diagnosis in both groups was UTI, mainly caused by E. coli. A similar proportion of blood and urine cultures were performed, whereas lumbar puncture was more frequently performed in SARS-CoV-2 negative infants (40.2% vs 16.9%, *p* = 0.001).

**Conclusions:**

Although the risk of concomitant serious bacterial infection with SARS-CoV-2 is low, it remains non-negligible. Therefore, even in SARS-CoV-2-positive febrile infants, we suggest that the approach to screening for SBIs remains cautious.

**Supplementary Information:**

The online version contains supplementary material available at 10.1186/s13052-024-01721-x.

## Introduction

Fever in children is one of the most common causes of medical evaluation, especially in pediatric emergency department (PED) visits [1, 2]. Most of these febrile episodes are caused by self-limiting viral illnesses [3]. However, due to the lack of vaccination protection and immaturity of their immune systems, infants younger than 90 days of age are at higher risk of severe and invasive bacterial infections (SBIs and IBIs). The latter group includes bacteremia, sepsis and meningitis. SBIs account for approximately 5–15% of cases [4], of which 2 to 4% are IBIs [5].

Clinical signs and symptoms of viral and bacterial infections in young infants are frequently similar, making it challenging to identify patients with SBIs based only on the clinical presentation alone. In addition, several studies have shown that the risk of SBIs remains not negligible even in the presence of positive point-of-care viral tests [6, 7]. Therefore, febrile young infants often undergo extensive and invasive diagnostic procedures, followed by empiric antibiotic therapy, especially in the first month of life.

Many efforts have been made to develop risk stratification tools to early identify infants with SBIs and IBIs, based on specific predictors such as clinical appearance, age, and laboratory test results [8–10]. These tools have shown high sensitivity, although lower specificity.

During the COVID-19 pandemic, total PED visits decreased due to reduced transmission of common viral infections. However, some studies reported an increase in the number of SBIs and IBIs during the pandemic [11]. Even if the number of SBIs and IBIs was higher in infants without Sars-CoV-2 infection, about 10% of patients with COVID-19 infection were eventually diagnosed with IBIs [6]. Early identification of febrile young infants at risk of SBIs or IBIs in the pandemic era became even more challenging.

Our study aimed to evaluate whether the proportion of SBIs and IBIs in febrile infants younger than 90 days during the COVID-19 pandemic was higher than that in the pre-pandemic period and to describe the proportion of SBIs and IBIs in infants with and without SARS-CoV-2 infection.

## Methods

### Study design, setting and population

This was a retrospective single-center cohort study conducted at the Children's Hospital of the University of Padua in Italy, involving febrile young infants evaluated in the Pediatric Emergency Department (PED) and admitted to the Pediatric Acute Care Unit (PACU) or Pediatric Intensive Care Unit (PICU) between March 2017 to December 2022. Our Children's Hospital provides primary and secondary care to a metropolitan area of 350,000 people, 45,000 below 15 years of age, and tertiary care for a regional and extra-regional population, with 25,000 to 27,000 PED visits annually.

The study included infants less than 90 days of age, with fever at home or at the evaluation in the PED, independently of the reported or documented accompanying signs and symptoms.

Infants without fever, even in the presence of infectious diseases such as bronchiolitis or upper respiratory tract infections, were not included in the study.

The study cohort was stratified by age into four different groups (0–21 days, 22–28 days, 29–60 days, 61–90 days), reflecting the risk-stratification used by the American Academy of Pediatrics guideline on the management of febrile young infants (AAP guidelines [5]).

The study period was divided into two distinct timeframes: pre-pandemic, from March 2017 to February 2020, and pandemic, from March 2020 to December 2022. Each period was divided into sub-periods of twelve months, from March to February, except for the final period, which only covered ten months.

### Definitions

A Serious Bacterial Infection (SBI) was defined as the growth of a bacterial pathogen in a sterile body site, such as urine, blood, stool, or cerebrospinal fluid (CSF). A urine culture was considered positive in case of growth of more than 10,000 colony-forming units/ml in samples collected through transurethral bladder catheterization, more than 50,000 colony-forming units in case of clean void urine, associated in all cases with the presence of white blood cell at the urine analysis.

An Invasive Bacterial Infection (IBI) was defined as the growth of a bacterial pathogen in the blood or CSF.

### Data collection

Data were manually collected from electronic medical records using a password-protected REDCap data collection form. In particular, we abstracted clinical data about signs and symptoms preceding the medical evaluation and developing during the admission, laboratory and imaging investigation results, microbiological data, including SARS-CoV-2 swabs, and blood, urine, and CSF culture results.

Data abstractors (GG and FR) received formal training in medical records review but were not blinded to the study objectives. Periodic meetings with the study coordinators (GB, SR, SB) were held to discuss conflicting, ambiguous or missing data as well as review of inclusion and exclusion criteria. We followed recommended strategies for retrospective data abstraction [12].

All data were stored on the secure server at Padua University. To guarantee anonymity and privacy, each patient was assigned a unique study-specific identifier.

The Padua University Hospital Institutional Review Board approved the study (Protocol Number 306n/AO/22).

### Outcomes

The primary outcome was to compare the proportion of SBIs or IBIs in all febrile young infants assessed in our PED between the pre-pandemic and pandemic years.

The secondary outcome was to compare the proportion of SBIs and IBIs in infants with and without SARS-CoV-2 infection during the pandemic period. Furthermore, in this sub-group analysis, the proportion of infants that underwent sepsis workup and the proportion of different bacteria isolated were compared between the SARS-CoV-2 positive and negative groups.

### Statistical analysis

Results were summarized as numbers and percentages (categorical variables) and median and interquartile range. Categorical variables were compared with χ2 or Fisher's 2-tailed exact test in a contingency table, and continuous variables were compared with the non-parametric Kruskal–Wallis rank-sum test.

The proportions of infants with SBIs and IBIs were calculated in each sub-period to evaluate differences between the pre-pandemic and pandemic periods, and a χ2 statistic was calculated. Furthermore, the proportion of SBIs and IBIs in infants with and without SARS-CoV-2 infection during the pandemic were calculated to evaluate the differences between the two groups using the χ2 test.

Data were analyzed using Stata/MP 4 version 18 [13].

## Results

From March 2017 to December 2022, 442 febrile infants younger than 90 days were evaluated in Padua PED and admitted to the ward, per internal guidelines on managing fever in this age group.

### Comparison between pre-pandemic and pandemic years

Table 1 summarizes infants' epidemiological and clinical characteristics in the two cohorts. Demographic characteristics were similar across the study periods. There was an increasing, although not significant, trend of infants with at least one comorbidity during the pandemic period, especially between 2021 and 2022. Overall, SARS-CoV-2 positive infants were 71 (16.1%).

**Table 1:** Demographic characteristics, diagnostic testing SARS-CoV-2 infection of febrile neonates and infants 90 days or younger, before and during the COVID-19 pandemic

		**Total (*n* = 442)**		**Prepandemic cohort**						**Pandemic cohort**						***p*-value**
				**Mar 2017- Feb 2018(*n* = 82)**		**Mar 2018- Feb 2019(*n* = 65)**		**Mar 2019- Feb 2020(*n* = 102)**		**Mar 2020-Feb 2021(*n* = 46)**		**Mar 2021-Feb 2022(*n* = 79)**		Mar 2022- Dec 2022*(*n* = 68)		
		**No**	**(%)**	**No**	**(%)**	**No**	**(%)**	**No**	**(%)**	**No**	**(%)**	**No**	**(%)**	**No**	**(%)**	
Gender	Female	187	(42.3)	35	(42.7)	29	(44.6)	40	(39.2)	18	(39.1)	34	(43.0)	31	(45.6)	0.958
	Male	255	(57.7)	47	(57.3)	36	(55.4)	62	(60.8)	28	(60.9)	45	(57.0)	37	(54.1)	
Age group (days)	0–21	121	(27.4)	27	(32.9)	16	(24.6)	26	(25.5)	14	(30.4)	18	(22.8)	20	(29.4)	0.613
	22–28	56	(12.7)	11	(13.4)	12	(18.5)	15	(14.7)	4	(8.7)	6	(7.6)	8	(11.8)	
	29–60	181	(41.0)	26	(31.7)	26	(40.0)	47	(46.1)	17	(37.0)	38	(48.1)	27	(39.7)	
	61–90	84	(19.0)	18	(22.0)	11	(16.9)	14	(13.7)	11	(23.9)	17	(21.5)	13	(19.1)	
	Median (IQR)	35	(20–55)	30	(18–57)	34	(22–54)	33	(21–50)	36.5	(17–58)	43	(24–60)	34	(17–50)	0.333
Comorbidities	None	370	(83.7)	71	(86.6)	57	(87.69)	89	(87.3)	37	(80.4)	61	(77.2)	55	(80.9)	0.670
	One	62	(14.0)	10	(12.2)	7	(10.77)	10	(9.8)	7	(15.2)	16	(20.3)	12	(17.7)	
	Two or more	10	(2.3)	1	(1.2)	1	(1.54)	3	(2.9)	2	(4.3)	2	(2.5)	1	(1.5)	
Premature birth		24	(5.5)	4	(5.0)	4	(6.2)	5	(4.9)	3	(6.5)	4	(5.1)	4	(5.9)	0.998
SARS-CoV-2	Positive result	71	(16.1)	n.a	-	n.a	-	n.a	-	11	(23.9)	25	(31.6)	35	(51.5)	

**Fig. 1:**
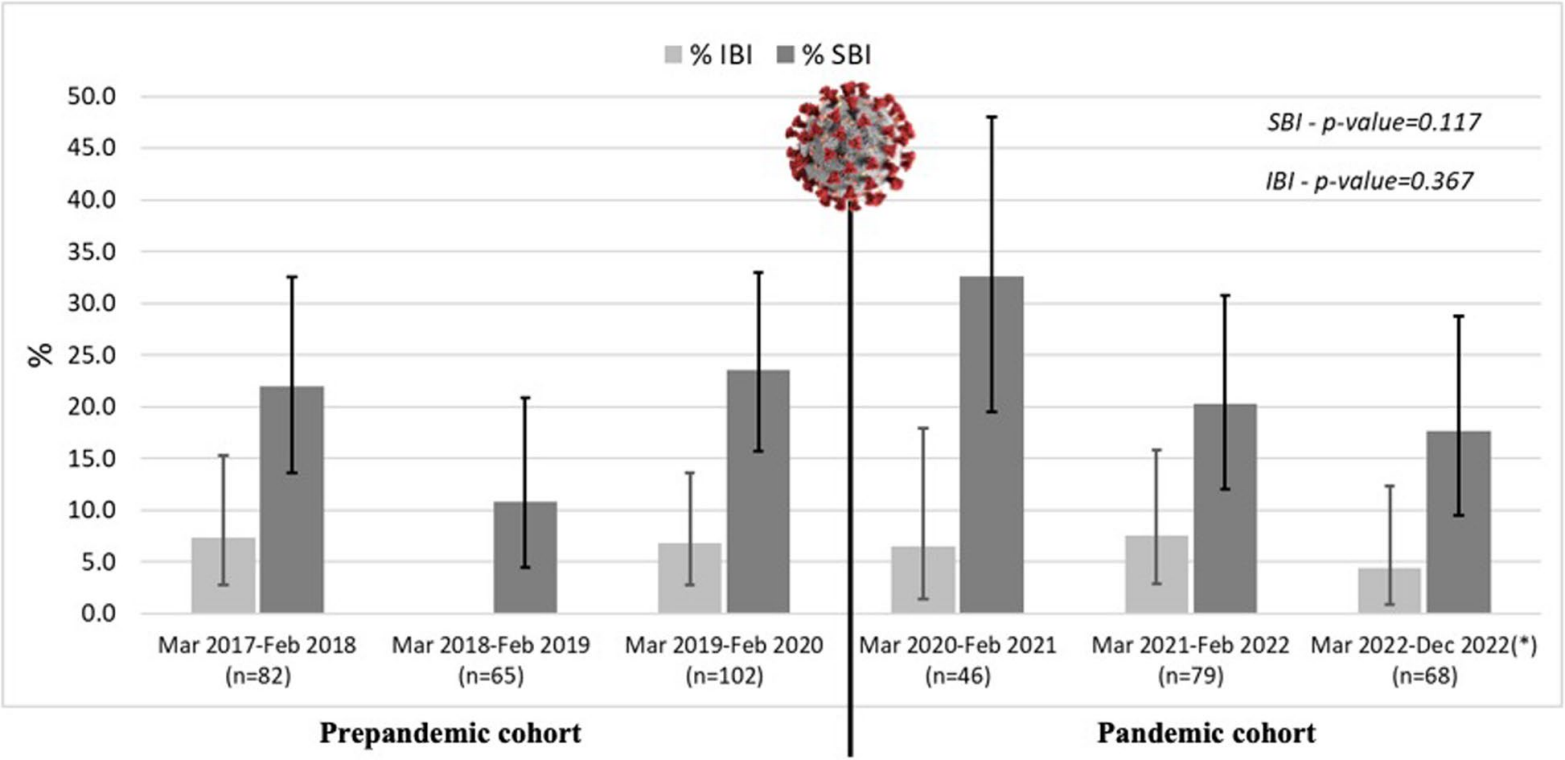
Proportion of severe and invasive bacterial infection before and during the COVID-19 pandemic. Legend: IBI, Invasive Bacterial Infection; SBI, Severe Bacterial Infection; n, number; *the last period only covered ten months

The proportion of SBIs did not significantly change over the study periods, ranging between 10.8% and 32.6% (*p* = 0.117). The slight increase in percentage observed in the first year of the pandemic is associated with the lowest recorded number of admitted patients (denominator) (Fig. 1). Considering the discharge diagnosis, the most frequent SBI was urinary tract infection (UTI), representing an overall 21.5% of diagnoses (Supplementary Table 1). A breakdown of SBI percentage by study period and age group is reported in Supplementary Table 2. The frequency of SBI was higher in the first 28 days of life.

The proportion of IBIs was also similar over the study periods, ranging between 0% and 7.6% (*p* = 0.367). Considering the discharge diagnosis, the most frequent IBI reported was sepsis (one child was discharged with a diagnosis of suspected sepsis due to clinical presentation and blood test results but had no microbiological confirmation and, for this reason, was not considered in the IBI group). A higher number of bacterial meningitis was observed in the pandemic years.

### Comparison between SARS-CoV-2 positive and negative infants

Demographic and clinical characteristics of patients analyzed by SARS-CoV-2 status are described in Table 2.

**Table 2:** Demographic characteristics of febrile neonates and infants 90 days or younger during the COVID-19 pandemic period (March 2020-December 2022) by the result of the SARS-CoV-2 test

		**Total(*n* = 193)**		**SARS-CoV-2 positive(*n* = 71)**		**SARS-CoV-2 negative(*n* = 122)**		***p*-value**
		**No**	**(%)**	**No**	**(%)**	**No**	**(%)**	
Gender	Female	83	(43.0)	37	(52.1)	46	(37.7)	0.051
	Male	110	(57.0)	34	(47.9)	76	(62.3)	
Age group (days)	0–21	52	(26.9)	11	(15.5)	41	(33.6)	0.050
	22–28	18	(9.3)	8	(11.3)	10	(8.2)	
	29–60	82	(42.5)	41	(57.7)	41	(33.6)	
	61–90	41	(21.2)	11	(15.5)	30	(24.6)	
	Median (IQR)	39	(19–57)	40	(27–53)	35	(16–60)	0.784
Comorbidities	None	153	(79.3)	58	(81.7)	95	(77.9)	0.338
	One	10	(5.2)	10	(14.1)	25	(20.5)	
	Two or more	3	(1.6)	3	(4.2)	2	(1.6)	
Premature birth		11	(5.7)	3	(4.2)	8	(6.6)	0.500

The median age and the presence of comorbidities were similar between the two patient groups. The prevalence of males was slightly higher in SARS-CoV-2 negative infants (62.3% vs 47.9%, *p* = 0.051).

**Fig. 2:**
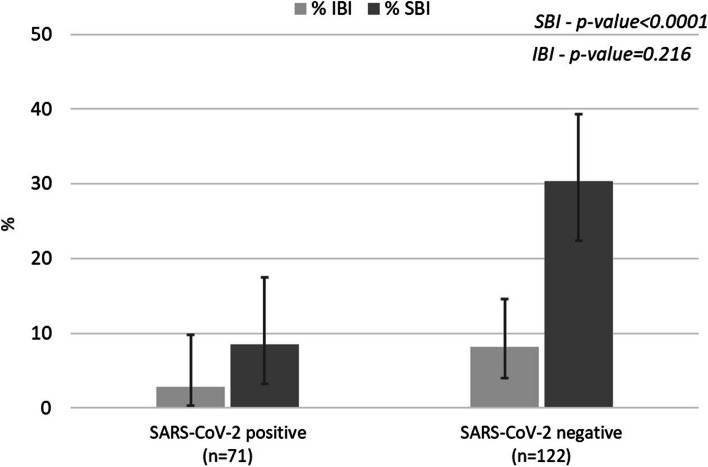
Proportion of severe and invasive bacterial infection during the COVID-19 pandemic period, by SARS-CoV-2 results. Legend: IBI, Invasive Bacterial Infection; SBI, Severe Bacterial Infection; n, number

The proportion of infants with a diagnosis of SBI was higher in the SARS-CoV-2 negative group (30.3%) compared to the positive group (8.5%), with a statistically significant result (*p* < 0.0001) (Fig. 2). UTIs were diagnosed in 32.8% of infants in the negative group and in 9.9% in the positive group (*p* < 0.0001). IBIs were less frequent, representing 8.2% of the diagnoses in infants with fever in the negative group compared to 2.8% in the positive group. No bacterial meningitis was recorded in SARS-CoV-2 positive infants (Supplementary Table 3). A breakdown of SBI percentage by SARS-CoV-2 status and age group is reported in Supplementary Table 4.

**Fig. 3:**
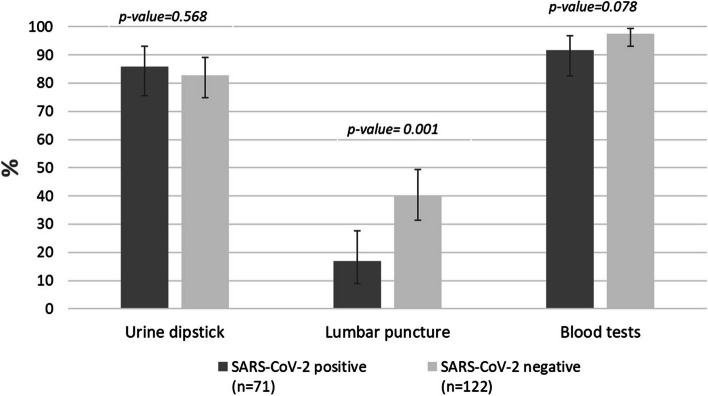
Diagnostic procedures in febrile infants during the COVID-19 pandemic, by SARS-CoV-2 results. Legend: IBI, Invasive Bacterial Infection; SBI, Severe Bacterial Infection; n, number

A similar proportion of blood and urine cultures were performed in SARS-CoV-2 positive and negative patients. A lumbar puncture was more frequently performed in SARS-CoV-2 negative infants (40.2% vs 16.9%, *p* = 0.001) (Fig. 3).

Results of blood, urine, and CSF cultures by SARS-CoV-2 are described in Supplementary Tables 5 and 6. The most common bacteria isolated from blood cultures were contaminants (Coagulase-negative Staphylococcus), representing 39% of all positive blood cultures.

E. coli was the most common bacteria isolated in urine culture, representing 68% of all positive urine cultures.

A CSF culture was performed on all infants who successfully underwent lumbar puncture and was negative in all infants in the SARS-CoV-2 positive group, while three pathogens and one contaminant were isolated in the SARS-CoV-2 negative group (Supplementary Table 6).

## Discussion

Our study found that the percentage of SBIs and IBIs in febrile young infants presenting to the PED although subject to fluctuation, did not significantly change over time during the pre and post-pandemic period. The slight increase in SBIs percentage in the first year of the pandemic was determined by a decrease in the overall number of infants presenting with fever, thus affecting the denominator, rather than an increase in SBIs absolute frequency. This is in line with a recent study reporting a significant decrease in the number of PED visits during the COVID-19 pandemic in the Veneto region [14] due to the reduction of viral infections, as reported by other reports [15, 16].

The SARS-CoV-2 positive group was less frequently affected by SBIs, particularly UTIs. However, the frequency of UTIs in these patients was relevant (approximately 10%), supporting the performance of a urine test in all febrile SARS-CoV-2 positive young infants. Although the percentage of IBIs was lower in the SARS-CoV-2 positive group, and no case of bacterial meningitis was detected, the small numbers do not allow for accurate estimates of IBI frequency in both patient groups.

Our results are in line with those already published. A recent study, published in 2023 by Aronson et al. [17], conducted on more than 14 thousand infants, showed that infants with SARS-CoV-2 infection were at a lower risk of SBI. However, still 1% of patients with SARS-CoV-2 infection had an SBI, with UTI being the most frequent (0.8%). These results are confirmed by a recent systematic review on the topic published by Pérez-Porra et al. [6]. This meta-analysis included 33 studies and analyzed the risk of having an IBI stratified by age. The estimated pool prevalence of IBI was 0.14% (95%CI 0.02%-0.027). Stratified by age, the prevalence of IBI was higher in the neonatal period (0.56%, 95%CI 0.0%-1.27%, in those 0–21 days old and 0.53%, 95%CI 0.0%-1.22%, in those 22–28 days old) than in the older infants (0.11%, 95%CI 0.0–0.24%, in those 29–60 days old). For infants older than 28 days, blood tests could be avoided. However, the author concluded that urine dipsticks should always be performed to exclude urinary tract infections.

In our study, only two out of 71 SARS-CoV-2 positive infants had a diagnosis of IBI, both with sepsis, one caused by E. coli and one by Enterococcus, and none had meningitis.

Nevertheless, in contrast with previously published studies, our study did not find statistically significant differences in the overall proportion of patients diagnosed with SBI between the pre-pandemic period and the pandemic years. In the study by Mittal et al. [11], published in January 2022 and considering data until February 2021, the proportion of infants with SBI increased from 11.7 and 6.9% (in 2018 and 2019, respectively) to 28.4% in 2020. Like in our study the explanation behind the increase described in this article lies in the reduction in the number of children evaluated in the PED for fever and acute respiratory tract infections. Indeed, strict public health strategies to prevent the spread of SARS-CoV-2 infection reduced the circulation of other infectious agents, especially viruses [18, 19]. Consequently, young infants evaluated for fever in the PED during the first year of the pandemic had a lower risk of having a viral infection and a higher risk of having SBI.

Our study demonstrated a similar pattern, with an increase in the proportion of infants with SBIs observed between March 2020 and February 2021. However, subsequent years, which were not assessed by Mittal et al., exhibited a decrease in the proportion of patients with SBI, resuming levels seen in the pre-pandemic years. In the latter half of 2021, the COVID-19 containment measures were relaxed, leading to the resumption of children attending school and kindergarten. Consequently, viral infections, particularly Respiratory Syncytial Virus, began to circulate again, resulting in an anticipated peak and the number of PED evaluations and admissions of infants with fever surged again [20, 21]. This change in viral epidemiology did not determine a lower frequency of SBIs. Furthermore, although some studies reported reduced SBI in febrile infants with other viral respiratory infections, coinfection remains possible and non negligible [7].

The number of infants infected by SARS-CoV-2 increased throughout the COVID-19 pandemic period, from 23.9% between March 2020 and February 2021 to 51.5% in the ten months between March 2022 and December 2022, during which more than half of patients evaluated and admitted for fever were SARS-CoV-2 positive. As reported in the literature, infants and children were less frequently infected by SARS-CoV-2 in the first pandemic than in the subsequent waves [22].

Considering SBI, our study showed that the proportion of infants with a diagnosis of SBI and positive for SARS-CoV-2 was 10%, lower than SARS-CoV-2 negative patients (for whom we observed an SBI rate above 30%) but not negligible.

In line with other studies published [17, 23, 24], the most common diagnosis was UTI, with E. coli as the most frequently isolated bacterium in each group.

Our study supports the results already published, which showed that infants with SARS-CoV-2 have a lower, but non-negligible, risk of SBI [6, 25–29]. This underscores the importance of adhering to the American Academy of Pediatrics (AAP) guidelines, which recommend a comprehensive sepsis workup—including blood tests, blood cultures, urine cultures, and lumbar punctures—for all infants under 21 days old, regardless of their SARS-CoV-2 test results. This approach ensures that any potential bacterial infection or, although rare, herpes virus infection is promptly identified and treated, preventing severe complications. While our study does not provide sufficient data to stratify the risk of SBI based on precise age brackets, we observed a trend suggesting that well-appearing infants older than 28 days who tested positive for SARS-CoV-2 may have a lower risk of SBI and IBI. Nevertheless, it remains crucial to perform a urine dipstick test on all these infants to rule out urinary tract infections, which are common in this age group. For infants older than 28 days presenting with symptoms consistent with COVID-19, current evidence suggests that it may be possible to avoid other invasive procedures such as lumbar punctures, provided there are no clinical finding to suspect a SBI. COVID-19 in this age group has not been associated with severe clinical manifestations and outcomes; therefore, in case of ill appearance or severe clinical presentation, IBI and SBI should be taken into account [30]. This approach is supported by other studies [6, 31], which highlight that non-invasive testing can be sufficient in certain clinical scenarios. This helps to reduce the stress and potential complications associated with invasive procedures, while still ensuring appropriate and timely care for these infants.

Our study has some limitations. First, due to the study's retrospective nature, not all the information was always available in the electronic charts. Second, this is a single-center study, and the number of infants analyzed is small. Furthermore, it was not possible to collect data in 2023, limiting the last sub-period of our study to ten months and making it impossible to evaluate the complete re-opening after the COVID-19 pandemic, with the resurgence of all viral infections, including the Influenza virus.

## Conclusion

Although the risk of concomitant serious bacterial infection with SARS-CoV-2 infection is low, it remains non-negligible. Therefore, even in SARS-CoV-2 positive febrile infants, we suggest that the approach to screening for SBIs remains cautious, following the guidance provided by the American Academy of Pediatrics, especially in infants younger than 30 days of age evaluated with fever.

### Supplementary Information

Supplementary Material 1

## Data Availability

The data used in this study cannot be made publicly available due to Italian data protection laws. The anonymized datasets generated during the current study can be provided on request, from the corresponding author, after written approval by the local ethic committee.

## Abbreviations

AAP: American Academy of Pediatrics
CSF: Cerebrospinal Fluid
IBI: Invasive Bacterial Infection
PACU: Pediatric Acute Care Unit
PED: Pediatric Emergency Department
SBI: Severe Bacterial Infection
UTI: Urinary Tract Infection
