# Supplementary material for: Severe and invasive bacterial infections in infants aged less than 90 days with and without SARS-CoV-2 infection
Source: Ital J Pediatr. 2024 Aug 15;50:148. doi: 10.1186/s13052-024-01721-x (PMC11325733; doi:10.1186/s13052-024-01721-x)
Supplement: Supplementary file 1 — Supplementary Material 1 [file 13052_2024_1721_MOESM1_ESM.docx]

Table 1S – Discharge diagnosis of febrile neonates and infants 90 days or younger before and during the COVID-19 pandemic by age group

|  |  | **Total (n=442)** | | **Prepandemic cohort** | | | | | | **Pandemic cohort** | | | | | | **pvalue** |
| --- | --- | --- | --- | --- | --- | --- | --- | --- | --- | --- | --- | --- | --- | --- | --- | --- |
|  |  |  |  | **Mar 2017-**  **Feb 2018 (n=82)** | | **Mar 2018 –**  **Feb 2019 (n=65)** | | **Mar 2019 –**  **Feb 2020 (n=102)** | | **Mar 2020 –**  **Feb 2021 (n=46)** | | **Mar 2021 -Feb 2022 (n=79)** | | **Mar 2022 –**  **Dec 2022 (n=68)** | |  |
|  |  | **No.** | **(%)** | **No.** | **(%)** | **No.** | **(%)** | **No.** | **(%)** | **No.** | **(%)** | **No.** | **(%)** | **No.** | **(%)** |  |
| **Sepsi//bacteremia susptected or confirmed** |  | **21** | **(4.82)** | **5** | **(6.1)** | **1** | **(1.5)** | **7** | **(6.7)** | **1** | **(2.2)** | **5** | **(6.3)** | **2** | **(2.9)** | 0.538 |
| Age group (days) | *0-21* | *10* |  | *1* |  | *0* |  | *4* |  | *0* |  | *3* |  | *2* |  |  |
|  | *22-28* | *3* |  | *0* |  | *1* |  | *2* |  | *0* |  | *0* |  | *0* |  |  |
|  | *29-60* | *6* |  | *3* |  | *0* |  | *0* |  | *1* |  | *2* |  | *0* |  |  |
|  | *61-90* | *2* |  | *1* |  | *0* |  | *1* |  | *0* |  | *0* |  | *0* |  |  |
| **Urinary Tract Infections** |  | **95** | **(21.5)** | **16** | **(19.5)** | **11** | **(16.9)** | **21** | **(20.6)** | **16** | **(34.8)** | **16** | **(20.2)** | **15** | **(22.1)** | 0.312 |
| Age group (days) | *0-21* | *27* |  | *8* |  | *3* |  | *4* |  | *2* |  | *7* |  | *3* |  |  |
|  | *22-28* | *8* |  | *2* |  | *1* |  | *1* |  | *1* |  | *1* |  | *2* |  |  |
|  | *29-60* | *33* |  | *2* |  | *5* |  | *10* |  | *7* |  | *3* |  | *6* |  |  |
|  | *61-90* | *27* |  | *4* |  | *2* |  | *6* |  | *6* |  | *5* |  | *4* |  |  |
| **Meningitis** |  | **6** | **(1.4)** | **0** | **(0.0)** | **0** | **(0.0)** | **1** | **(1.0)** | **3** | **(6.5)** | **1** | **(1.3)** | **1** | **(1.5)** | 0.063 |
| Age group (days) | *0-21* | *4* |  | *0* |  | *0* |  | *0* |  | *2* |  | *1* |  | *1* |  |  |
|  | *22-28* | *0* |  | *0* |  | *0* |  | *0* |  | *0* |  | *0* |  | *0* |  |  |
|  | *29-60* | *2* |  | *0* |  | *0* |  | *1* |  | *1* |  | *0* |  | *0* |  |  |
|  | *61-90* | *0* |  | *0* |  | *0* |  | *0* |  | *0* |  | *0* |  | *0* |  |  |

**Table 2S -** Age distribution (number and percentage) of febrile neonates and infants 90 days or younger with severe bacterial infection before and during the COVID-19 pandemic

|  |  | **Total (n=92)** | | **Prepandemic cohort** | | | | | | **Pandemic cohort** | | | | | | **pvalue** |
| --- | --- | --- | --- | --- | --- | --- | --- | --- | --- | --- | --- | --- | --- | --- | --- | --- |
|  |  |  |  | **Mar 2017-Feb 2018 (n=18)** | | **Mar 2018- Feb 2019 (n=7)** | | **Mar 2019- Feb 2020 (n=24)** | | **Mar 2020 -Feb 2021 (n=15)** | | **Mar 2021 -Feb 2022 (n=16)** | | **Mar 2022 -Dec 2022 (n=12)** | |  |
|  |  | **No.** | **(%)** | **No.** | **(%)** | **No.** | **(%)** | **No.** | **(%)** | **No.** | **(%)** | **No.** | **(%)** | **No.** | **(%)** |  |
| Age group (days) | 0-21 | 31 | (33.7) | 9 | (50.0) | 2 | (28.6) | 6 | (25.0) | 3 | (20.0) | 7 | (43.7) | 4 | (33.3) | 0.946 |
|  | 22-28 | 6 | (6.5) | 1 | (5.6) | 1 | (14.3) | 1 | (4.2) | 1 | (6.7) | 1 | (6.3) | 1 | (8.3) |  |
|  | 29-60 | 29 | (31.5) | 4 | (22.2) | 2 | (28.6) | 10 | (41.7) | 6 | (40.0) | 4 | (25.0) | 3 | (25.0) |  |
|  | 61-90 | 26 | (28.3) | 4 | (22.2) | 2 | (28.6) | 7 | (29.2) | 5 | (33.3) | 4 | (25.0) | 4 | (33.3) |  |

**Table S3** – Discharge diagnosis of febrile neonates and infants 90 days or younger during the COVID-19 pandemic period (March 2020-December 2022) by age group and the result of the SARS-CoV-2 test

|  | | **Total**  **(n=193)** | | **SARS-CoV-2 positive**  **(n=71)** | | **SARS-CoV-2 negative**  **(n=122)** | | **pvalue** |
| --- | --- | --- | --- | --- | --- | --- | --- | --- |
|  | | **No.** | **(%)** | **No.** | **(%)** | **No.** | **(%)** |  |
| **Sepsi//bactermia suspected or confirmed** | | **8** | **(4.2)** | **2** | **(2.8)** | **6** | **(4.9)** | **0.713** |
| *Age group (days)* | *0-21* | *5* |  | *1* |  | *4* |  |  |
|  | *22-28* | *0* |  | *0* |  | *0* |  |  |
|  | *29-60* | *3* |  | *1* |  | *2* |  |  |
|  | *61-90* | *0* |  | *0* |  | *0* |  |  |
| **Urinary Tract Infections** | | **47** | **(24.4)** | **7** | **(9.9)** | **40** | **(32.8)** | **<0.0001** |
| *Age group (days)* | *0-21* | *12* |  | *1* |  | *11* |  |  |
|  | *22-28* | *4* |  | *1* |  | *3* |  |  |
|  | *29-60* | *16* |  | *5* |  | *11* |  |  |
|  | *61-90* | *15* |  | *0* |  | *15* |  |  |
| **Meningitis** | | **5** | **(2.6)** | **0** | **(0.0)** | **5** | **(4.1)** | **0.160** |
| *Age group (days)* | *0-21* | *4* |  | *0* |  | *4* |  |  |
|  | *22-28* | *0* |  | *0* |  | *0* |  |  |
|  | *29-60* | *1* |  | *0* |  | *1* |  |  |
|  | *61-90* | *0* |  | *0* |  | *0* |  |  |

**Table S4 -** Age distribution (number and percentage) of febrile neonates and infants 90 days or younger with severe bacterial infection, positive and negative at the SARS-CoV-2 test

|  | | **Total**  **(n=43)** | | **SARS-CoV-2 positive**  **(n=6)** | | **SARS-CoV-2 negative**  **(n=37)** | | **pvalue** |
| --- | --- | --- | --- | --- | --- | --- | --- | --- |
|  | | **No.** | **(%)** | **No.** | **(%)** | **No.** | **(%)** |  |
| Age group (days) | 0-21 | 14 | (32.6) | 2 | (33.3) | 12 | (32.4) | 0.214 |
|  | 22-28 | 3 | (7.0) | 1 | (16.7) | 2 | (5.4) |  |
|  | 29-60 | 13 | (30.2) | 3 | (50.0) | 10 | (27.0) |  |
|  | 61-90 | 13 | (30.2) | 0 | (-) | 13 | (35.1) |  |

**Table S5** – Blood and urine cultures of febrile neonates and infants 90 days or younger during the COVID-19 pandemic period (March 2020-December 2022) stratified based on the results of SARS-CoV-2 swab

|  | | **Total**  **(n=193)** | | **SARS-CoV-2 positive**  **(n=71)** | | **SARS-CoV-2 negative**  **(n=122)** | | **pvalue** |
| --- | --- | --- | --- | --- | --- | --- | --- | --- |
|  | | **No.** | **(%)** | **No.** | **(%)** | **No.** | **(%)** |  |
| **Blood culture** | |  |  |  |  |  |  | 0.387 |
| Not performed | | 22 | (11.4) | 10 | (14.1) | 12 | (9.8) |  |
| Failed procedure | | 4 | (2.1) | 3 | (4.2) | 1 | (0.8) |  |
| Performed - negative | | 153 | (79.3) | 53 | (74.6) | 100 | (82.0) |  |
| Performed – not available | | 1 | (0.5) | 0 | (-) | 1 | (0.8) |  |
| Performed - positive |  | 13 | (6.7) | 5 | (7.0) | 8 | (6.6) |  |
| *CoNs* | | *5* |  | *2* |  | *3* |  |  |
| *E. coli* | | *2* |  | *1* |  | *1* |  |  |
| *Streptococcus agalactiae* | | *2* |  | *0* |  | *2* |  |  |
| *Enterococchi* | | *1* |  | *1* |  | *0* |  |  |
| *S. aureus* | | *1* |  | *0* |  | *1* |  |  |
| *Klebsiella pneumoniae* | | *1* |  | *0* |  | *1* |  |  |
| *Kocuria rhizophila* | | *1* |  | *1* |  | *0* |  |  |
| **Urine culture** | |  |  |  |  |  |  | 0.001 |
| Not performed | | 51 | (26.4) | 23 | (32.4) | 28 | (23.0) |  |
| Failed procedure | | 4 | (2.1) | 4 | (5.6) | 0 | (-) |  |
| Performed - negative | | 72 | (37.3) | 30 | (42.3) | 42 | (34.4) |  |
| Performed – not available | | 1 | (0.5) | 0 | (-) | 1 | (0.8) |  |
| Performed - contaminated | | 12 | (6.2) | 5 | (7.0) | 7 | (5.7) |  |
| Performed - positive |  | 53 | (27.5) | 9 | (12.7) | 44 | (36.1) |  |
| *E. coli* | | *36* |  | *6* |  | *30* |  |  |
| *Klebsiella pneumoniae* | | *5* |  | *0* |  | *5* |  |  |
| *Enterococchi* | | *2* |  | *0* |  | *2* |  |  |
| *Citrobacter* | | *2* |  | *0* |  | *2* |  |  |
| *Enterobacter aerogenes* | | *2* |  | *1* |  | *1* |  |  |
| *Klebsiella oxytoca* | | *4* |  | *2* |  | *2* |  |  |
| *s. Agalactiae* | | *1* |  | *0* |  | *1* |  |  |
| *Other* | | *0* |  | *0* |  | *1* |  |  |

**Table S6** – Microbiological tests on CSF of febrile neonates and infants 90 days or younger during the COVID-19 pandemic period (March 2020-December 2022) stratified based on the results of SARS-CoV-2 swab

|  | | **Total**  **(n=56)** | | **SARS-CoV-2 positive**  **(n=13)** | | **SARS-CoV-2 negative**  **(n=43)** | |
| --- | --- | --- | --- | --- | --- | --- | --- |
|  | | **No.** | **(%)** | **No.** | **(%)** | **No.** | **(%)** |
| **Film-array panel on CSF** | |  |  |  |  |  |  |
| Not performed | | 2 | (3.6) | 2 | (15.4) | 0 | (-) |
| Performed - negative | | 41 | (73.2) | 10 | (76.9) | 31 | (72.1) |
| Performed - positive |  | 13 | (23.2) | 1 | (7.7) | 12 | (27.9) |
| *Enterovirus* | | *7* |  | *0* |  | *7* |  |
| *Streptococcus agalactiae.* | | *2* |  | *0* |  | *2* |  |
| *HHV-6* | | *2* |  | *1* |  | *1* |  |
| *E. coli* | | *1* |  | *0* |  | *1* |  |
| *Other* | | *1* |  | *0* |  | *1* |  |
| **CSF culture** | |  |  |  |  |  |  |
| Failed procedure | | 3 | (5.4) | 3 | (23.1) | 0 | (-) |
| Performed - negative | | 49 | (87.5) | 10 | (76.9) | 39 | (90.7) |
| Performed - positive |  | 4 | (7.1) | 0 | (-) | 4 | (9.3) |
| *Streptococcus agalactiae* | | *2* |  | *0* |  | *2* |  |
| *E. coli* | | *1* |  | *0* |  | *1* |  |
| *Staphylococcus hominis* | | *1* |  | *0* |  | *1* |  |
